# Enablers and barriers to dietary change for Māori with nutrition-related conditions in Aotearoa New Zealand: a scoping review

**DOI:** 10.1017/S136898002400212X

**Published:** 2024-10-22

**Authors:** Christina McKerchar, Christine Barthow, Tania Huria, Bernadette Jones, Kirsten J. Coppell, Rosemary Hall, Tutangi Amataiti, Amber Parry-Strong, Soana Muimuiheata, Morag Wright-McNaughton, Jeremy Krebs

**Affiliations:** 1 Department of Population Health, University of Otago, Christchurch, New Zealand; 2 Department of Medicine, University of Otago, Wellington, New Zealand; 3 Department of Māori Indigenous Health Innovation, University of Otago, Christchurch, New Zealand; 4 Centre for Endocrine, Diabetes and Obesity Research (CEDOR), Wellington, New Zealand; 5 Total-Wellbeing Consultancy Ltd, Auckland, New Zealand; 6 Formerly of Department of Medicine, University of Otago, Wellington, New Zealand

**Keywords:** Nutrition therapy, Māori people, Aotearoa New Zealand, Indigenous peoples, Dietary change

## Abstract

**Objective:**

Māori, the Indigenous population of Aotearoa New Zealand, face a substantial burden of nutrition-related diseases, especially obesity and type 2 diabetes. Weight loss, through dietary change, is a central component of obesity and diabetes prevention and management; however, most approaches have not been designed with or evaluated specifically for Māori. The aim of this study was to review literature on the enablers and barriers to dietary change, for Māori.

**Design:**

Relevant literature published from January 2000 to May 2024 was identified by searches in Medline (Ovid), Embase (Ovid), Scopus, Indigenous health (informit), CINAHL (EBSCO), Web of Science and NZResearch. Studies included Māori and reflected enablers and barriers to dietary change for individuals/whānau (families). Data identifying the aims, methods, interventions, location, population studied and identified enablers and barriers to dietary change and responsiveness to Māori were extracted. Enablers and barriers to dietary change were mapped to a New Zealand Indigenous health framework, the Meihana model.

**Setting:**

Settings included studies based in Aotearoa New Zealand, where participants were free living and able to determine their dietary intake.

**Participants:**

Studies included at least 30 % Māori participants.

**Results:**

Twenty-two of the seventy-seven identified records met the inclusion criteria. Records included a diverse range of research approaches.

**Conclusions:**

Using a relevant Indigenous model, this study highlights that multiple and diverse enablers and barriers to dietary change exist for Māori and the critical importance of developing interventions, in close partnership with Indigenous communities, grounded in Indigenous understandings of health.

Dietary interventions are complex and the ability to sustain changes to dietary habits is a critical factor in the effectiveness of dietary interventions for chronic conditions such as type 2 diabetes (T2DM). Multiple micro-level factors, such as health literacy, self-discipline, stress, family and social support, and macro-level factors, such as socio-economic deprivation, cultural factors and the food environment, influence the outcomes of those attempting to modify their dietary habits^(1,2)^. Like many Indigenous populations internationally, all of these are highly relevant to Māori, the Indigenous people of Aotearoa New Zealand (NZ). Within such communities, understanding the historical context and the enablers and barriers to dietary change is important.

Māori are tangata whenua or people of the land. Māori history in Aotearoa dates to approximately 1300 AD when ancestors migrated to Aotearoa from the Eastern Polynesian Islands of the Pacific. Māori developed a unique culture adapted to the natural environment of Aotearoa, sourcing food from the sea, rivers, wetlands and forests and cultivating foods such as kumara or sweet potato^(3)^.

In 1840, the Treaty of Waitangi, an agreement between the chiefs of many Māori tribes and the British Crown, was signed^(4)^. It was drafted in English and then translated into a Māori version known as Te Tiriti o Waitangi. Te Tiriti o Waitangi consisted of three articles. In Article 1, Māori acknowledged British ‘kawanatanga’, the right of governance. In Article 2, Māori retained ‘rangatiratanga’ with the promise to uphold the authority that tribes had always had over their lands and taonga (treasures). In Article 3, the Crown promised Māori the benefits of royal protection and full citizenship^(4)^. This article obliges the Crown to positively promote equity, including equitable access to the determinants of health such as housing, education and food security to ensure equitable health outcomes^(5)^.

Following the signing of the treaty, colonisation caused widespread loss of land and political reorganisation, dramatically impacting all aspects of Māori life, including health and food^(6)^. Initially, Māori adapted to introduced European food sources, such as potatoes, pigs and wheat, and cultivated and traded these with European migrants and participated in the early NZ economy^(7)^. This changed dramatically post-1860 with the outbreak of war and invasion of Māori tribal areas to secure land for European settlement^(8)^. The subsequent alienation of land by the colonial government through a variety of different mechanisms left Māori virtually landless in their own country by the early 1900s^(3)^. Without land to provide an economic base, many Māori became impoverished and unable to build intergenerational wealth^(6)^.

In the newly established colony, little consideration was given to Māori conceptualisations of the environment. Wetlands, rivers and harbours that had traditionally provided sources of food were drained for farmland, rubbish dumps or sewage discharge^(9)^. Large parts of the forest were cleared, introduced pests devastated native bird populations and conservation estates were created that did not consider the ways in which Māori used native plants and animals for food and medicine. The rapid urbanisation of the Māori population from the 1940s impacted communal ways of living and connection to tribal areas^(10)^. This means that many Māori have been unable to undertake customary food practices, which has impacted the transmission of mātauranga and access to healthy food and contributes to poor health outcomes on every front. The food environment in NZ today is obesogenic, with relatively cheap, heavily promoted unhealthy foods widely available^(11)^, and Māori dietary patterns are now reflective of a globalised food supply. Additionally, many Māori whānau (extended families) and an estimated one-third of Māori children experience food insecurity^(12)^.

Today, Māori face a substantial burden of nutrition-related diseases, especially overweight, obesity and T2DM. One in three NZ adults is obese, and the rates are higher for Māori (48 %)^(13)^. In 2021, the estimated rate of diabetes was higher amongst Māori (7 %) than the national average (4·2 %)^(14)^. This rate is impacted by socio-economic deprivation, with those living in high deprivation areas experiencing the greatest burden^(14)^. This is exacerbated by the widespread marketing and availability of unhealthy foods in lower socio-economic areas^(15)^. Weight loss is an essential part of obesity management and the prevention and optimal management of T2DM^(16)^. A range of dietary approaches may be effective in achieving weight loss^(17)^ and improving health; however, most have not been designed with or evaluated specifically for different cultural groups, particularly for Māori^(1)^. There have been two relatively recent systematic reviews on interventions to prevent and manage obesity in Māori adult and child populations; however, these both combined Māori and Pacific data and were not solely related to dietary approaches but to obesity more broadly^(18,19)^. There is also a review about how Māori navigate nutrition advice, but this is focused specific on nutrition advice and does not directly discuss enablers and barriers of dietary change for Māori^(20)^.

Therefore, the main objective of this scoping review was to systematically locate and review the literature on the enablers and barriers to dietary change for Māori. Second, this review sought to evaluate the extent to which the selected literature reported how Māori interests were represented throughout the research. Finally, the implications for future research, policy and service development were considered.

## Methods

Scoping reviews utilise systematic searches and mapping of literature to produce a synthesis of evidence about what is already known and identify gaps in the literature. Given the uncertainty about what is already known on this topic, this approach is suitable to address the aims of this project^(21)^. Our methods were informed by recommendations from the Johanna Briggs Institute^(22,23)^ and Pollock *et al.* ^(21)^. The review was pre-registered on the Open Science Framework (https://doi.org/10.17605/OSF.IO/AJCXK) and is reported according to the Preferred Reporting Items for Systematic Reviews and Meta-Analyses extension for Scoping Reviews^(24)^ (see online supplementary material, Supplemental Table S1). Our original intent was to include both Māori and Pacific peoples in this analysis; however, after the initial search for data, we undertook separate reviews for each of these ethnic groups. The research team included Māori (CM, BJ, TH), Pacific (TA, SM) and Pākehā (non-Māori) (CB, KC, RH, AP-S, MW-M, JK,) researchers. In this review, the perspectives of Māori were prioritised. From the outset, the team agreed to adopt the give way rule, whereby if any cultural differences in interpretations of data occurred, the views of the researchers holding Māori understanding would predominate^(25)^.

### Analysis

**Figure 1.**
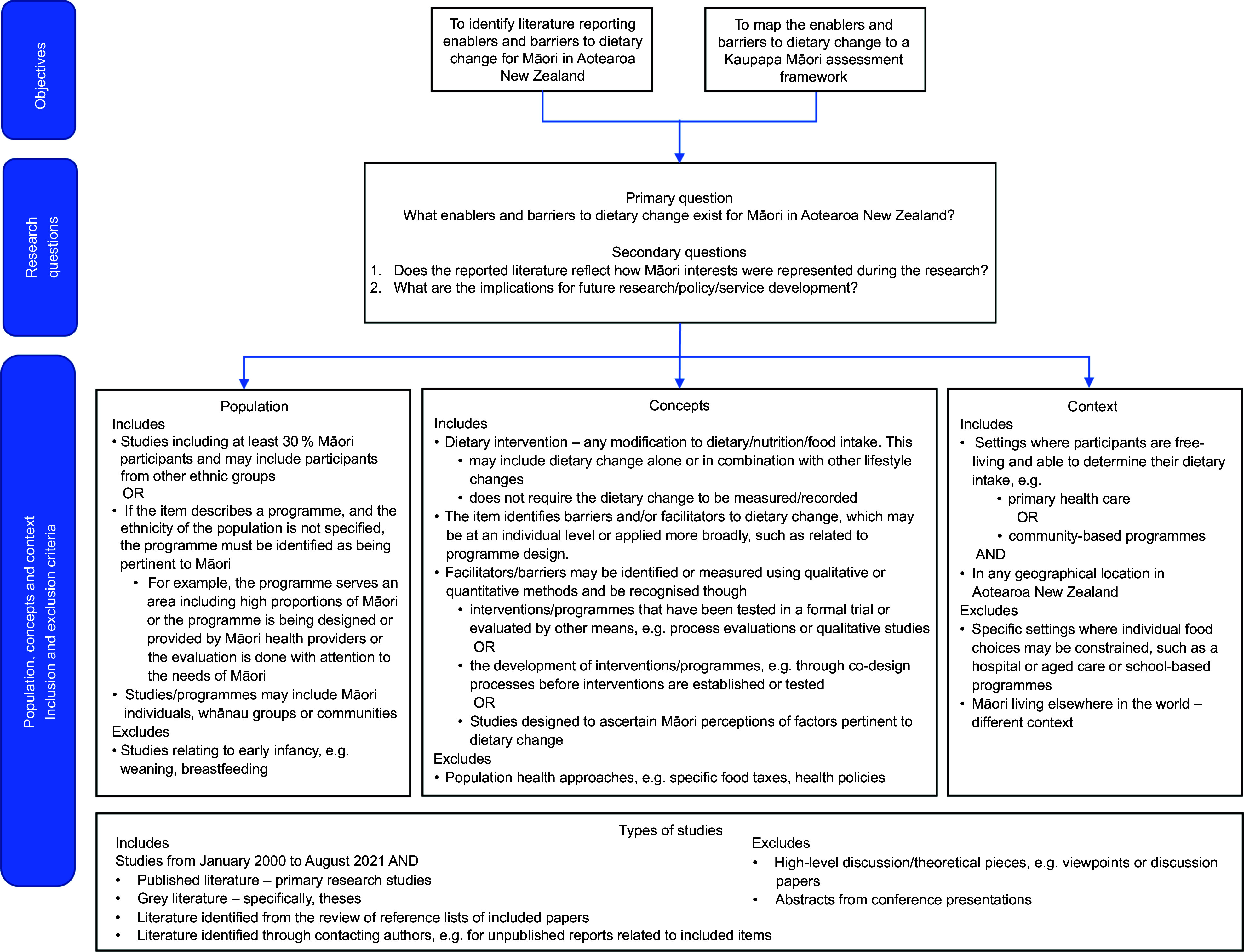
Summary of scoping review objectives, questions, population, concepts, context and inclusion and exclusion criteria.

The research objectives, questions, definitions used to define the population, concepts and context and inclusion and exclusion criteria are depicted in Fig. 1 ^(22)^. Record identification, mapping and analysis were completed in sequence as indicated in Table 1. For the purposes of this review, we modified definitions used in a previous study by KC and defined enablers as those factors that prompted people to want to make a dietary change and/or participate in programmes aimed at dietary change, and barriers were those factors that inhibited the adoption of dietary change^(28)^. We aimed to assess the records included in this review in relation to their relevance to Māori by using the CONSIDER criteria that recommend^(26)^ reporting should cover multiple domains including partnership and governance, research prioritisation, relationships with stakeholders and participants, researcher expertise related to Indigenous health, methodological approach including attention to factors related to colonisation, racism, sociocultural and economic context, participation, capacity building, how data analysis and interpretation reflect Indigenous values and strength-based approaches and finally how the dissemination of findings facilitates Indigenous advancement^(26)^. These CONSIDER statement criteria are designed to ensure that the reporting of health research monitors Indigenous participation, knowledge and priorities and reduces the potential for research to be used as a tool of colonisation^(29)^.

**Table 1.** Sequence and methods for identifying and selecting records and analysis

Search strategies (see Supplementary files) utilising MESH or subject terms/key words were completed in Medline (Ovid), Embase (Ovid), Scopus, Indigenous health (informit), CINAHL (EBSCO), Web of Science and NZResearch identified relevant literature published from January 2000 to August 2021. This timeframe was selected to include research over 20 years reflecting contemporary food environments and when Indigenous cultural knowledge has become more widely accepted. Title and abstract screening of the literature was completed in duplicate (MM-W and AP-S or AP-S and CB) using Covidence systematic review software (Veritas Health Innovation). Full-text screening applying the criteria in Fig. 1 was undertaken (CM, CB). All differences were resolved by consensus with no requirement for the give way rule^(25)^. Reference lists of selected records were checked for further relevant literature (CB). The study aims, methods, interventions, location, population studied and enablers and barriers to dietary change were extracted into a Microsoft Excel spreadsheet independently and in duplicate for five studies (CM and CB). After confirming the consistency of coding, data for the remaining selected records were extracted by either reviewer with the other person checking the data. The extent to which studies were likely to reflect Māori concerns was assessed using the 2019 CONSolIDated critERia for strengthening the reporting of health research involving Indigenous peoples (CONSIDER) criteria^(26)^. However, the record reporting did not align to the CONSIDER criteria. Therefore, we extracted data reflecting a range of items relevant to responsiveness to Māori as recorded in Table 2. Finally, the enablers and barriers were mapped using an Indigenous Māori theoretical framework called the Meihana model^(27)^, which is detailed below. Coding of five papers was undertaken independently (CM, CB), findings were compared, then any uncertainties about coding were discussed with one of the model authors (TH) and the coding was modified accordingly. The remainder of coding was completed by either coder and cross-checked (CM, CB). Relevant search strategies were repeated to cover the period from August 2021 to May 2024, and no new literature meeting the inclusion criteria was identified. This was with the removal of search terms related to Pacific peoples as we have undertaken a separate review for Pacific people, and this analysis is focused on Māori.

### The Meihana model

**Figure 2.**
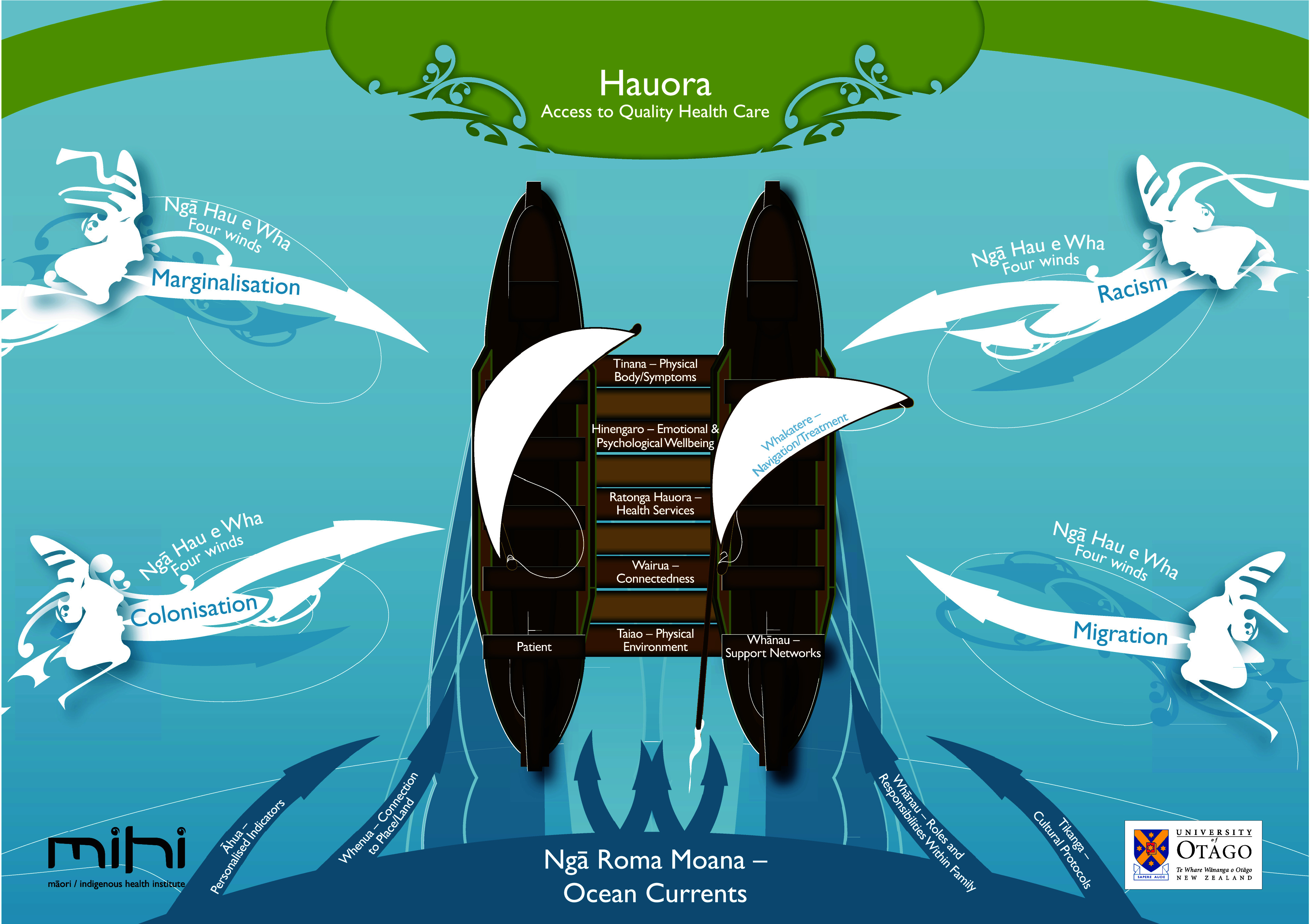
Diagram of the Meihana model. (This figure was originally published in Pitama S, Huria T, Lacey C. Improving Māori health through clinical assessment: Waikare o te Waka o Meihana. NZMJ. 2014;127(1393):107–19. Reproduced with permission from NZMJ).

The Meihana model is an Indigenous health framework developed at the University of Otago, Christchurch, to assist health practitioners to work effectively with Māori^(27)^. The Meihana model has been used in teaching^(30)^ to improve cultural competency and within health professions, for example, by clinical psychologists^(31)^. It has also been used as a theoretical framework in research^(32)^. A diagram of the Meihana model is presented in Fig. 2, and a description of its components is in Table 2. We use the domains of the Meihana model for our analysis and interpretations and to present the results of this scoping review.

**Table 2.** Description of the Meihana model

This Meihana model includes four components and emphasises that clinical assessment should include or analyse the following components: **Waka hourua (double-hulled canoe)** is representative of the patient and their whānau (extended family) Whānau (family) support systems Tinana or a patient’s physical status including diet and exercise Hinengaro – their psychological and emotional well-being Wairua – their beliefs regarding connectedness and spirituality Taiao – the physical environment of a patient/whānau Ngā Ratonga Hauora – health services and systems **Ngā Hau e Wha (the four winds)** is a metaphor describing four interconnected factors that negatively impact the journey of the waka hourua towards well-being hauora or well-being for Māori. Colonisation: This aspect challenges health practitioners to explore factors such as poverty, including how food insecurity has been impacted by the context of New Zealand history. It also asks health practitioners to consider how stereotyping may impact bias in clinical decisions. Racism: This recognises the discrimination that exists across the health system and acknowledges racism as a determinant of health that can impact Māori health and engagement with health services and health professionals. Migration: The internal migration of Māori from their turangawaewae (place of belonging) in largely rural areas to other urban areas of New Zealand may impact access to support networks and land. Marginalisation: The forced adoption of a Western-dominant culture led to a loss of Indigenous knowledge and Māori becoming a minority population. Health professionals need a good understanding of health information including current Māori health status, health disparities and health gains. **Ngā Roma Moana (ocean currents)** cultural factors that can impact Māori on their journey towards hauora. Ahua – personal indicators of Te Ao Māori – validation of Māori identity within a clinical setting Tikanga Māori – cultural principles – each patient/whānau will have specific cultural principles that impact aspects of life Whānau – the relationships, roles and responsibilities of the patient within Te Ao Māori (Māori world view) including whānau, hapū (subtribe), iwi (tribe) and other organisations. Whenua – specific genealogical or spiritual connection between patient/whānau and land **Whakatere (navigation)** is the importance of navigation and plotting a course for proposed best practice treatment, intervention and management.

## Results

### Description of included records

**Figure 3.**
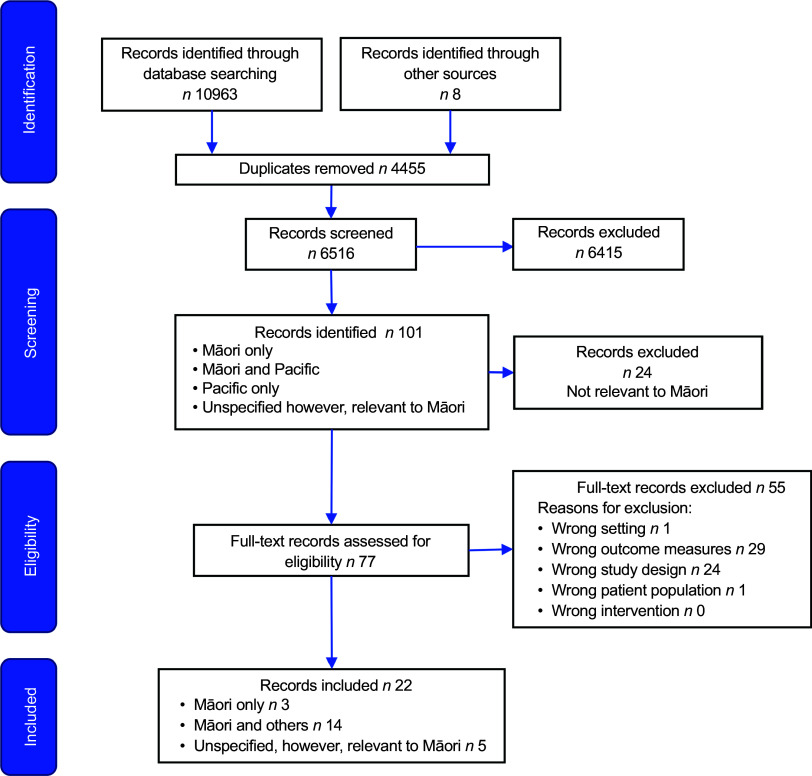
Flow chart of record selection.

Figure 3 details the selection of twenty-two records from seventy-seven full-text records reviewed for inclusion in this review. Table 3 shows that of the twenty-two records, three sets reported one or more different aspects of the same primary studies, and this resulted in a total of fifteen independent studies being included in the review. Participants recruited were all Māori for only three of the fifteen studies^(35,36,53)^. Fourteen records related to findings for Māori as well as other ethnicities (see Table 3) with the proportion of Māori in each study ranging from 31 to 87 %. A further five records did not document participants’ ethnicity but were included based on their relevance to Māori: the development of a community programme for Māori^(46)^, Māori health provider programme development or evaluations^(48–50)^ or the co-design of a programme for Indigenous and other groups^(52)^. Where specified, records reported on studies including participants aged 3–70 years. The records reported on interventions that were directed towards those with prediabetes, T2DM, overweight/obesity and multiple long-term conditions. In some cases, records included an index participant and their whānau or caregivers or communities (see Table 3). Dietary change was typically one component of a broader lifestyle change programme in most studies. The research aims and methodological approaches varied considerably, and the records were categorised into four groups: (a) enablers/barriers to dietary change directly assessed through an intervention focused on individuals, (b) research that sought to understand perspectives or experiences of Māori rather than a specific intervention and therefore enablers and barriers were inferred, (c) direct assessment of enablers/barriers related to an implemented programme and (d) a mixed group of records describing a programme or resource development and in some cases components of evaluation that utilised methods, where barriers and enablers to dietary change might be inferred or directly assessed (see Table 3).

**Table 3.** Description of included records and responsiveness to Māori

First author, Year, Reference	Aims	Methodology	Population	Responsiveness to Māori
Directly assesses enablers/barriers of an individual-focused intervention				
**Description of intervention:** Pilot study of a 6-month primary care-based nurse-led intervention with six components including (1) 10 h culturally appropriate nurse training with dietitian support, monthly case reviews with dietitian and liaison nurse support; (2) individual patient education including dietary assessment, personalised goal setting and dietary advice sessions; (3) consistent messages and opportunistic reminders provided by general practitioners; (4) nutritionally supportive primary care environment; (5) community-based group education for patients and whānau – six weekly sessions, 1–1·5 h each; and (6) written patient resources **Setting:** Urban general practice				
Coppell, 2017^(33)^	To examine patient and provider perspectives on the implementation, feasibility and acceptability of a 6-month multi-level primary care nurse-led prediabetes lifestyle intervention	Convergent mixed methods with qualitative process evaluation Direct observation, meeting notes and semi-structured interviews with key informants and participants Thematic analysis	*n* 157 Ethnicity, 31 % Māori Gender, 48 % Female Age range, adults ≤ 70 years Conditions, overweight/obese with prediabetes	Appropriate Māori cultural advice provided, and advisor is one of study authors Practices with high proportion Māori patients included in study Close attention paid to cultural etiquette when interviewing Participants quotes identified by ethnicity
Abel, 2018^(34)^	To explore the experiences of people recently diagnosed with prediabetes in making dietary changes following a 6-month primary care nurse-delivered dietary intervention pilot	Qualitative study Semi-structured interviews Thematic analysis	*n* 20 Ethnicity, 45 % Māori Gender, 50 % Female Age range 43–69 years Conditions, overweight/obese with prediabetes	Appropriate Māori cultural advisor for study Non-Māori interviewer; however, close attention was paid to cultural etiquette when interviewing Māori and Pacific people Study identifies Māori specific finding in managing social pressures around food Good explanation of manaakitanga in discussion
**Description of intervention:** Fully powered study of 6-month prediabetes intervention package including (1) primary care nurse training and support from a local dietitian; (2) individualised dietary assessment, goal setting and dietary advice taking into account socio-economic and cultural influences; (3) key messages and consistent opportunistic reminders; (4) nutritionally supportive primary care environment; (5) community-based group education for participants and their family; and (6) written patient resources **Setting:** Urban general practices				
Abel, 2021^(28)^	To understand motivators, enablers and challenges to dietary change amongst a diverse sample of New Zealanders with prediabetes participating in a primary care nurse-led individualised dietary intervention	Qualitative study	*n* 58 Ethnicity, 48 % Māori Gender, 52 % Female Age range 28–69 years Conditions, adults with prediabetes and BMI ≥ 25 kg/m^2^	Appropriate Māori cultural advice provided, and advisor is one of study authors Interviewer supported by Māori researcher Aimed for 50 % Māori in sample Māori cultural oversight in analysis Coding and analysis were undertaken systematically and separately for subgroups by gender, ethnicity
Understand individual’s perspectives/experiences – Enablers/barriers are inferred				
Description of intervention: No intervention Setting: Urban				
Arthur, 2017^(35)^	To investigate how low carbohydrate, high-fat diets are perceived by members of the Māori community and the barriers and facilitators to following this type of diet	Questionnaire and qualitative study Individual interviews focused on perceptions of a specific dietary pattern Inductive thematic analysis	*n* 18 Ethnicity, 100 % Māori Gender, 72 % Female Age range 25–65 years Conditions, no specific criteria	Master’s thesis candidate is of Māori/Samoan descent Supervisors Māori Sole focus of study is on Māori; however, approach is not explicitly stated
Description of intervention: No intervention Setting: Not stated				
Bell, 2017^(36)^	To elicit understandings of obesity in Māori	Qualitative study informed by Indigenous knowledge systems Semi-structured interviews in Te Reo, Māori Thematic analysis	*n* 15 Ethnicity, 100 % Māori Gender, 53 % Female Age range 25–56 years Conditions, BMI ≥ 30 kg/m^2^	Lead author is Māori supported by Māori researchers and elders Study informed by Indigenous knowledge framework Interviews in Te Reo (Māori language) Indigenous knowledge acquisition Ensured engagement aligned with and responsive to Indigenous epistemologies Strongly centred in Te Ao Māori - methods and interpretation
Description of intervention: No intervention Setting: Not stated				
Francis, 2018^(37)^	To report role of food and mealtimes and effect of nutritional advice on lives of people with several long-term conditions	Multiple qualitative case studies Multiple conversations and two semi-structured interviews Inductive thematic analysis using Te Whare Tapa Whā model as explanatory framework	*n* 16 Ethnicity, 38 % Māori Gender, 56 % Female Age range 26–88 years Conditions, multiple long-term conditions	Non-Māori research team Advice provided throughout by two Kaumātua who also assisted with recruitment Analysis using lens of Te Whare Tapa Whā Participants quotes identified by ethnicity
Description of intervention: No intervention Setting: Urban				
Glover, 2019^(38)^	To explore Māori parents/caregivers’ views of the importance of weight to health, and the enablers and barriers to a healthy weight in children aged 6 months to 5 years, and decision-making regarding food provision	Grounded qualitative study Focus group data from Māori focus groups only (however, these included some Pacific) Content-driven inductive thematic analysis	*n* 40 Ethnicity, 87 % Māori Gender, 89 % Female Age range > 20 years Conditions, parents/caregivers of children aged 6 months to 5 years	Lead Māori researcher Ethnically matched focus group facilitator Māori researcher analysis
Directly assesses enablers/barriers of an implemented programme				
**Description of intervention:** Whānau Pakari programme 12-month multidisciplinary (coordinator, dietitian, physical activity coordinator, psychologist and paediatrician) home and community-based intervention programme for children and adolescents with obesity and their whānau. Initial home visit with dietitian and physical activity coordinator. Forty weekly activity and education sessions include physical activity/psychology/dietitian sessions, supermarket tours, healthy food on a budget, portion sizes and vegetable gardens over 12 months. Interventions culturally tailored, for example, traditional Māori games, recipes adapted for Māori. Whānau linked to local activity centre at conclusion of the intervention period **Setting:** Urban-rural setting, community based				
Anderson, 2018, (Chapters 8 and 15)^(39)^	To develop and assess multidisciplinary intervention for children and adolescents with obesity and ensure the programme is accessible for Māori and other derived groups. Determine the outcomes of the programme and its acceptability to stakeholders, participants and referrers	Mixed methods including RCT and process evaluation of the programme satisfaction assessing including referrers to programme, providers and Māori and other ethnic end users	*n* 290 index participants Ethnicity, 37 % Māori, index participant Gender, 50 % Female Age range 3·4–16·1 years for index participant Conditions, index participant overweight BMI > 91^st^ centile with co-morbidities or obese BMI > 98^th^ centile Process evaluation participant feedback *n* 8 children, 25 % Māori *n* 6 family members, 33 % Māori	PhD thesis – ethnicity of candidate not stated De medicalised whānau and home-based multidisciplinary programme established after extensive consultation including Māori from multiple organisations and tribes within the community Name of programme (Whānau Pakari) gifted by Māori community representative Māori engagement and stakeholders – involved in service design, evaluation and feedback – an ongoing process Emphasis on relationship/partnership building, use of Indigenous staff, Indigenous knowledge and applying a Māori lens to service delivery (including relevant cultural adaptations) and curriculum Strengths based programme not focused on weight loss Participant focus group data collected by Māori researcher
Wild, 2021^(40)^	To identify challenges of making and sustaining healthy lifestyle changes for families with children/adolescents affected by obesity, referred to a multi-component healthy lifestyle assessment and intervention programme	Qualitative study, secondary analysis Semi-structured interviews with parents/caregivers of children/adolescents referred to a multidisciplinary childhood obesity programme Reflexive thematic analysis	*n* 42 Ethnicity, 33 % Māori Gender, 86 % Female Age range, not stated Conditions, parents/caregivers of obese children/adolescents	Senior Māori researcher contributed to research and checked manuscript Mixed Māori/non-Māori research team Māori interviews conducted by Māori interviewer with non-Māori interviewer present Ethnicity data collected using relevant protocols Koha offered as reciprocity for time Give way rule applied where final interpretations about Māori participants experiences rested with Māori researchers Participants quotes identified by ethnicity Method of feedback of results negotiated with participants and achieved by video
Wild, 2020^(41)^	To understand enablers and barriers to engagement in a multidisciplinary assessment and intervention programme for children and adolescents with obesity, particularly for Māori	Mixed methods survey with open-ended free text questions and Likert-type scales Likert scale presented by numbers and percentages Free text data – thematic analysis	*n* 71 Ethnicity, 45 % Māori Gender, 89 % Female Age range, not stated Conditions, parents, caregivers or children if aged over 11 years involved in multidisciplinary programme for children/adolescents with obesity	Mixed Māori/non-Māori research team Survey developed by Māori/non-Māori researchers and stakeholders Survey was informed by the framework of broader study which including Kaupapa Māori Questions designed to capture structural and organisational barriers and enablers rather than narrow focus on characteristics of individuals or families Flexible data collection options provided – online, phone survey or hardcopy by post Ethnicity data collected using relevant protocols Give way rule applied where final interpretations about Māori participants’ experiences rested with Māori researchers
Wild, 2020^(42)^	To understand the barriers and enablers to engagement in a multi-component assessment and intervention healthy lifestyle programme based in the home and community for children and their families	Qualitative study Semi-structured interviews Inductive thematic analysis	*n* 76 Ethnicity, 42 % Māori Gender, % Female, not stated Age range, not stated Conditions, parents, caregivers or children if aged over 11 years involved in multidisciplinary programme for children/adolescents with obesity	Mixed Māori/non-Māori research team Study design and research approach Informed by Kaupapa Māori theory Interviews with Māori led by Māori interviewer (implied rather than ethnicity stated explicitly) Ethnicity data collected using relevant protocols Koha given to acknowledge participant involvement Method of feedback of results negotiated with participants and achieved by video Give way rule applied where final interpretations about Māori participants’ experiences rested with Māori researchers
Wild, 2021^(43)^	To understand the barriers to and enablers of engagement for Māori in a community-based, assessment-and-intervention healthy lifestyle programme and ascertain the cultural appropriateness of the programme	Qualitative study Semi-structured interviews Thematic analysis	*n* 76 Ethnicity, 32 % Māori Gender, % Female, not stated Age range, not stated Conditions, parents, caregivers or children if aged over 11 years involved in multidisciplinary programme for children/adolescents with obesity	Mixed Māori/non-Māori research team Study design and research approach informed by Kaupapa Māori theory Interviews with Māori led by Māori interviewer (implied rather than ethnicity stated explicitly) Koha given to acknowledge participant involvement Study findings aligned to a model of racism and its and impact on engagement Give way rule applied where final interpretations about Māori participants experiences rested with Māori researchers Method of feedback of results negotiated with participants and achieved by video Manuscript reviewed by senior Māori researcher
Anderson, 2021^(44)^	To understand barriers and enablers to engagement for participants in a child and adolescent multidisciplinary programme for healthy lifestyle change programme	Qualitative study Focus groups guided by principles of Kaupapa Māori Thematic analysis	*n* 6 parents/caregivers Ethnicity, 33 % Māori Gender, % Female, not reported Age range, not stated Conditions, parents/caregivers of children/adolescents with obesity *n* 8 children/adolescents Ethnicity, 25 % Māori Gender, % Female, not reported Age range 4·5–12 years Conditions, Children/adolescents with obesity	Mixed Māori/non-Māori research team Kaupapa Māori approach Māori Researcher led focus groups. Analysis of data is strength based and informed by Kaupapa Māori research approach
**Description of intervention:** Co-designed, decolonised approach to supporting people living with poorly controlled T2DM and their whānau. Programme has three components: (1) a network hub; (2) Kai manaaki (skilled case workers) embedded in primary care work alongside patients and whānau to case manage care; and (3) a cross-sector network of services to whom whānau can be referred to address the wider determinants of health (Selak2018) **Setting:** Urban and rural				
Tane, 2021^(45)^	To explore how participants in the Mana Tū programme (a co-designed Māori-led diabetes support programme) construct and give meaning to their experiences navigating health and social services in Aotearoa, as well as their experiences living with T2DM and their journey in the Mana Tū programme	Qualitative study Kaupapa Māori approach Semi-structured interviews Inductive and deductive thematic analysis	*n* 22, Mana Tū participants, plus 10 whānau Ethnicity, 59 % Māori Gender, 59 % Female Age, mean 58 years Conditions, individuals with poorly controlled T2DM or whānau of individual	Mixed Māori/non-Māori team Programme is Māori led using Kaupapa Māori and whānau ora approaches Interviews conducted by Māori researcher Appropriate cultural etiquette followed for interviews Give way rule applied where final interpretations about Māori participants’ experiences rested with Māori researchers Quotes identify participant ethnicity
Describes programme/resource development and may include some aspects of evaluation – Enablers/barriers are inferred and/or directly assessed				
**Description of intervention:** No intervention – development phase only **Setting:** Urban				
Murphy, 2003^(46)^	To describe the process involved in developing a community programme for Māori and outline the novel aspects of the programme which contribute to the success	Description of programme development Focus group with programme participants Details and analysis and interpretation not reported	N/A	Lead author is Māori Mixed Māori/non-Māori research team Māori leadership involved in programme Strong engagement with local Māori over a prolonged period led by Māori members of the team Approvals gained from many Māori community groups Kaupapa Māori approach
**Description of intervention**: No intervention – resource development phase only **Setting:** Not stated				
Eyles, 2009^(47)^	To describe the development of six paper-based nutrition education resources for multi-ethnic participants in a large supermarket intervention trial.	Description of resource development using qualitative approach and focus groups General inductive thematic analysis	*n* 44 Ethnicity, 34 % Māori Gender, predominantly Female Age range 18–50 years Conditions, not reported	Mixed Māori/Pacific/non-Māori research team From study conception a research partnership was developed with Māori and Pacific community organisations Culturally matched focus group facilitators Participant quotes ethnicity identified Results considered by ethnicity
**Description of intervention:** Formative programme evaluation to improve health and well-being through health promotion activities. Components not specified in detail **Setting:** Small town/rural				
Henwood, 2007^(48)^	To improve health and well-being through health promotion programmes promoting healthy lifestyles based on an integrated kaupapa Māori framework	Community development action research – utilised as a formative evaluation of a programme Data from visits, hui, email, project document review and interviews Analysis and interpretation not specified	*n* 5 Māori health organisations Participants not described	Māori research group contracted to do formative evaluation of an iwi based project by the Ministry of Health Programmes based on holistic Kaupapa Māori framework and supported traditional Māori activities and knowledge transfer High level of Māori community involvement – ground-up development and capacity building in the community
**Description of intervention:** Project REPLACE promotes small achievable and regular changes over 12 weeks by replacing one unhealthy activity or choice with a healthier alternative. Core principles – regular exercise, eat healthy food, participate (reduce screen time), lose weight, alcohol reduction, smoking cessation and education to alter unhealthy thoughts. **Setting:** Six small community-based Māori health agencies from one region				
Mercer, 2013^(49)^	To evaluate and report Project REPLACE using the Kaupapa Māori lens	Short-term process and outcome evaluation Case study method using Kaupapa Māori perspective Data monthly reports, interviews focus groups, survey and direct observation	*n* 6 Māori health organisations Participants not described	Māori/non-Māori researchers Kaupapa Māori approach Māori team members did interviews and focus groups with Māori Qualitative as well as quantitative data used to ensure findings reflective of Māori world view of hauora (i.e. they didn’t just measure BMI)
Hamerton, 2014^(50)^	To illustrate an innovative health promotion programme aimed at improving Māori health and to discuss the importance of ownership and control of health initiatives by Māori	Kaupapa Māori evaluation of healthy eating, Healthy action programme Interview and focus group data from programme managers, coordinators participants and the wider community Thematic analysis	*n* 6 Māori health organisations providing HEHA programme Participants not described	Māori/non-Māori researchers Support throughout from a district health board cultural advisor Kaupapa Māori evaluation Data collection instruments used Māori models of health – Whare Tapa Whā Evaluation assessed broad community impact including iwi (tribe) and hapū (subtribe) impacts
**Description of intervention:** Culturally appropriate community-based 6-month weight loss competitions between teams with financial rewards **Setting:** Urban and rural				
Glover, 2021^(51)^	To report a culturally and community-based team intervention WEHI) trial on weight loss, healthy eating and physical activity in Māori and Pacific	Quasi-experimental study with statistical analysis Findings related to recruitment and retention discussed by authors	*n* 161 Ethnicity, 60 % Māori Gender, 80 % Female Age range > 16 years Conditions, BMI ≥ 30 kg/m^2^	Māori principal investigator and lead author Programme evaluated delivered by Ngati Hine Hauora Trust, Te Wakahuia Manawatu Trust and Pacific Heartbeat
**Description of intervention:** No intervention – development phase only **Setting:** North Island				
Verbiest, 2019^(52)^	To describe the co-design methods and processes used in the OL@-OR@ project. (a culturally tailored, behaviour change mHealth intervention for Indigenous and other priority communities)	Description of a co-designed project utilising partnerships and focus groups Data observation, photographs, transcripts Thematic analysis	N/A Co-design - partnership with Māori and other ethnic groups	Research team co-led by Māori, Pacific and European researchers Team representation includes Māori and Pacific providers Extensive time spent building partnerships, establish team and build capacity Extensive focus groups/other activities with relevant communities to determine their priorities and views throughout the process of designing a mHealth app Integration of Indigenous models of health
**Description of intervention:** Whānau-centred, community-based lifestyle programme 2–3 interactions per week over 8 weeks. Full programme details not specified but examples given. Ongoing changes made according to feedback **Setting:** Urban and rural community				
Masters-Awatere, 2021^(53)^	To discuss the co-design implementation and outcome evaluation of a whānau centred, community-based lifestyle programme Kimi ora) intended to ensure no worsening of HbA1c and to improve the well-being of Māori with diabetes/prediabetes	Community-based participatory approach and co-design Quantitative pre- and post-biological measures Qualitative interview and observational data Analysis process not detailed	*n* 35 Ethnicity, 100 % Māori Gender, 89 % Female Age range 30–69 years Conditions prediabetes, T2DM	Mainly Māori research team Uses community-based participatory research co-design approach with Māori providers

T2DM, type 2 diabetes; HbA1c, glycated Hb; RCT randomised controlled trial.

### Results mapped to Meihana model

#### Waka hourua (double-hulled canoe)

##### Whānau (support networks)

Whānau support was identified as a key enabler to encourage people towards adopting healthy eating and exercise habits^(28,37,45)^. Whānau would often adopt healthy habits together^(28)^, and this collective whānau approach to change was encouraged by many intervention studies^(42,53)^.

Conversely, dietary change could be a source of conflict within whānau, with whānau members either not supporting change or even undermining efforts towards change, for example, buying unhealthy foods^(28,34,37,46)^. A lack of household and whānau support was identified as a difficulty for some, such as solo parents coping with being the only adult in a household^(34)^. For children, inconsistent food patterns across different households within the same whānau could also impact their food options^(40)^.

##### Tinana (physical health and functioning of the patient)

Dietary change was not prioritised when there were other competing priorities to cope with such as other co-morbidities^(28)^. Many studies noted that having another chronic health condition such as sleep apnoea or depression impacted one’s motivation to change in people with diabetes or prediabetes^(28,34,37,46)^. In the case of children, other health issues such as neurodevelopmental disorders^(42)^ took precedence. Some parents described the taste preferences of children and the need to accommodate food allergies as also impacting dietary habits within families^(38)^. The difficulty of changing dietary habits was also noted^(47)^.

Low nutrition literacy and a lack of clear information about diet were identified as barriers impacting people’s/families’ ability to change^(35,38)^. One Indigenous co-designed intervention programme with a range of interventions, including increasing nutrition knowledge through week-to-week meal planning, nutritional label reading and discussion of alternatives to fast foods, did facilitate dietary changes, which resulted in improvements in weight, BMI and glycaemic control in participants^(53)^. Similarly, a primary care nurse-delivered prediabetes dietary intervention found that education to increase nutrition literacy such as understanding food labels or using frozen vegetables was an enabler of dietary change^(34)^.

##### Hinengaro (psychological and emotional well-being of the patient)

The diagnosis of diabetes or prediabetes was a psychological motivator for encouraging dietary change^(34,47)^, which was expressed as a desire to ‘be around’ for whānau, especially children and grandchildren^(34,47,52)^. Similarly, the determination not to get diabetes or develop the complications of diabetes, having seen the impact of diabetes on whānau members and wanting to ‘counteract’ a genetic predisposition towards obesity/diabetes, was a strong motivating factor for change^(28,34,35,42,45)^. The desire to be a role model within their own whānau was pertinent for some with patterns of intergenerational diabetes within their whānau^(45)^. One study identified rangatiratanga or empowerment as an important factor in facilitating change^(52)^.

Conversely, Tane *et al.* ^(45)^ discussed how the experience of T2DM had become normalised intergenerationally within some whānau, leading to a lack of motivation to change with people feeling that developing diabetes was inevitable. Other factors that undermined motivation included depression^(28,34)^, other co-existing mental health conditions^(40)^, feeling overwhelmed by the diagnosis^(45)^ or simply not feeling ready to make changes^(46)^. Two studies discussed the whakamā (shame) felt by people in not meeting the weight loss goals prescribed for them and how this led to a persistent sense of failure^(36,37)^. Francis *et al.* ^(37)^ also discussed that for people coping with long-term chronic conditions removing pleasure by not allowing preferred foods compounded their sense of loss of control.

##### Wairua (beliefs regarding connectedness and spirituality)

A spiritual connection to the land and the environment was described in some studies as a source of strength for participants^(36,52)^. One intervention, called ‘Korikori a Iwi’, was focused on improving physical activity and connected physical activity to taha wairua (spiritual health) by using the Māori language and traditional activities such as mau taiaha (martial arts) to encourage change^(48)^. Another study incorporated a goal related to spiritual health^(45)^. The value of connecting with whānau was evident in the development of OL@-OR@, a healthy lifestyle smart phone app for Māori: focus group participants identified whakapapa (ancestry) and mātauranga (traditional knowledge) as important enablers. In the resulting app, pictures and information about ancestral historical places were included, and users could also upload their own health related karakia (incantations)^(52)^.

##### Taiao (the physical environment of the patient/whānau)

The physical environment of a person and whānau both within households and within the wider community was identified as both an enabler and a barrier of change. A sense of not being able to control one’s household environment due to factors such as busyness, stress or being out of routine was noted in one study^(28)^. Visible signage around the community along with the availability and marketing of cheap unhealthy foods including at after-school events were also barriers^(28,38,40)^. In contrast, some interventions included having supportive healthy food environments, for example, having only healthy foods available at events^(49)^, having nutrition information available in waiting rooms^(33)^ and advocating for policy changes in schools, sports clubs and marae (Māori cultural centres)^(48)^.

An intervention setting was an important factor for community acceptance of an intervention. For example, Whānau Pakari (meaning ‘healthy, self-assured families that are fully active’) was delivered in a non-clinical setting at a regional sports trust, which was seen as effective in reaching and engaging with Māori^(34)^. Physical distance and inconvenient health service locations were also a barrier for Māori to access health care^(41)^.

##### Ngā Ratonga Hauora (health services and support systems)

Many studies focused on the role of health services and systems that provide support for patients/whānau with prediabetes or T2DM.

Studies emphasised the importance of approaching health from an Indigenous world view^(36,44,49)^ and focusing on a broader context including emotional, spiritual and relational health^(36,49)^ in relation to weight loss and dietary change. A narrow individualistic biomedical approach was seen as lacking an understanding of people’s lived realities, especially for those with co-morbidities^(36,37)^, and therefore culturally unsafe^(45)^. Some studies described people’s negative historical experiences in health services, as reinforcing weight stigma and discrimination^(40,44,45)^. Therefore, many studies^(37,40,41,43,45)^ emphasised the importance of culturally safe care where the focus was on relationship building, compassion and respect^(43)^ or the development of mutual understanding of language, cultural world view and sociocultural lived experience^(45)^. For one intervention, these were defined using Māori values: manaakitanga (the process of showing respect, support and care for others) and aroha (love, compassion, empathy, kindness)^(43)^. In the Mana Tū study (meaning ‘to stand with authority’), Kai Manaaki (community health navigators) were employed to attend health appointments and advocate for patients with the intention of disrupting an unequal power dynamic^(45)^.

A defining factor in contextualising health from an Indigenous world view was the importance of Māori leadership and engagement at a governance level of an intervention or programme^(39,45,46,48,53)^. This enabled Māori values such as collective family well-being or whānau ora^(45)^ to be privileged in programme design. Many studies discussed the involvement of the wider whānau within an intervention as an enabling factor for people to facilitate dietary changes^(44,47)^. One study noted that programmes aimed solely at an individual level often fail because food and eating are social practices and eating patterns form within groups^(37)^. From a practical viewpoint, one study noted the importance of childcare for whānau to attend intervention programmes^(38)^. As is discussed in the section on colonisation, a major barrier to dietary change for Māori is the impact of poverty. A further role of the community health navigators in the Mana Tū study was to facilitate access for participants to any welfare support they might be entitled to^(45)^.

A focus on ‘lifestyle’ rather than just weight loss was seen as a factor in the success of the Whānau Pakari intervention, which utilised a ‘demedicalised,’ family-friendly and community-focused approach^(39,44)^. The community-focused approach was also common across many studies that emphasised group support as a feature^(33–35,46,49)^. The group aspect of many programmes enabled people to develop strong supportive relationships with those with similar experiences and actively support each other, exchange ideas and strategies and motivate each other^(33,34,44,49)^.

A further feature of Māori specific interventions is that some drew from mātauranga Māori or Māori knowledge to encourage behaviour change. For example, the Korikori, a Iwi-based community physical activity intervention, encouraged exercise through using traditional Māori weaponry, kapa haka (Māori performing arts), waka ama (outrigger canoes), walking to historic sites and using marae (Māori cultural centres) as venues^(48)^. Similarly, Project REPLACE, a community-based lifestyle programme, encouraged activities such as Māori line dancing or seafood gathering to enhance the connection to whānau and hapū, foster togetherness and provide fun^(49)^. The Whānau Pakari intervention focused on providing a space where cultural aspirations were supported and identity respected^(44)^. They utilised the principle of whakamana (enabling of individuals and families) to support a family to become ‘self-determining’, in their process to achieve healthy lifestyle change^(44)^.

A key factor in the acceptance of interventions was the relationships formed between an individual/whānau and their healthcare team, for example, a group educator or primary healthcare nurse^(33)^. Participants valued the support and encouragement given by those working in primary health care^(28,34)^. In a 2003 study, participants valued a good relationship with the study dietitian and regularly being able to discuss food issues. They also valued trained staff with an understanding of behavioural change, dietary change and exercise^(46)^. The Whānau Pakari intervention was multidisciplinary and included a lifestyle coordinator, dietitian, physical activity coordinator and psychologist^(39,44)^. Strong effective relationships between those involved in an intervention delivery were also an important factor in their success^(33,39,44)^. This enabled easy referral processes and supported congruency in the advice given to participants^(48,50)^. Some highlighted a lack of system-level policy change in areas that impact the determinants of health for Māori^(40)^, which are discussed in the following section.

#### Ngā Hau e Wha (the four winds)

##### Colonisation

As outlined earlier, there are links between colonisation and food insecurity. For Māori, the cost of food and limited financial resources to afford healthy food were major barriers to making dietary changes^(28,33–35,38,46,47,52)^. These factors were evident along with a lack of time^(38,46,47,52)^ and in some instances a lack of knowledge about how to prepare healthy foods^(47)^. Large families were also an additional cost pressure for some^(47)^. Adverse stressful events also impacted participants’ ability to make dietary changes^(40)^. Participants were often living in ‘crisis’ mode or dealing with multiple challenges at home, including financial, food and housing insecurity, suicide, deaths in the family, mental health issues, disability and relocation^(40)^. One study highlighted historical trauma and the grief and shame of being culturally disenfranchised^(36)^, as a negative impact of colonisation for people, and another discussed the impact of colonisation on traditional foods, noting the impact of pollution on wild food sources^(35)^.

##### Racism

Few studies directly mentioned racism. In one study, it was noted that participation in the programme was impacted by institutional racism, which the authors linked to structural barriers, lower socio-economic conditions and interpersonal racism. Overall, these factors contributed to a distrust in the health system and therefore non-engagement^(42,43)^. Other authors also noted that people could be made to feel judged and inferior, leading to distrust^(45)^, or had experienced weight-based discrimination^(36)^, again impacting future engagement in health care.

##### Migration

Only two studies referred to the impact of migration on food. The separation of the land from the people was noted by Bell *et al.* ^(36)^, and Glover *et al.* ^(38)^ noted migration had contributed to a loss of food-growing knowledge and skill.

##### Marginalisation

This theme is about supporting health professionals in their understanding of current Māori health status and health gain. Most studies included in this review focused specifically on health issues inequitably experienced by Māori, and only three of the fifteen studies included Māori participants only^(35,36,53)^, and only one of these studies included an intervention, in the form of a community lifestyle programme^(53)^. A further three records evaluated two different Māori health provider programmes^(48–50)^.

#### Ngā Roma Moana (ocean currents)

The four elements identified in Ngā Roma Moana are the most common Te Ao Māori (Māori world view) concepts evident within clinical spaces that may impact the Māori experience of hauora. Therefore, studies that recognised these elements or explored these elements identified the important role they play for hauora.

##### Tikanga (Māori cultural principles)

Many studies discussed cultural expectations around providing and partaking of food, and in some cases, the feeling of pressure to eat food offered to avoid giving offence in social situations was challenging for people to navigate^(28,33,34,37,38)^. Two studies identified that the provision of healthy food options in cultural settings such as marae was an important enabler of healthy eating^(38,46)^. One study also discussed the importance of providing time to exercise at events^(46)^. Two intervention programmes discussed how they were able to embed changes into cultural situations to support healthy eating and physical activity at community events^(48,49)^.

##### Whānau (relationships, roles and responsibilities of the patient within Te Ao Māori)

This dimension is related to a person’s role and influence within their whānau. One study identified that a particular person could be the lead in terms of planning kai (food) for their whānau and therefore strongly influenced dietary change^(52)^. Other studies identified that taking on the role as kaumātua (Māori elder) and being able to still engage with mokopuna (grandchildren) motivated people towards dietary change^(28,35)^. Conversely, people’s roles within a whānau as carers either as time-poor parents^(38,42)^ or carers for hospitalised whānau members^(34)^ impacted their ability to make dietary changes.

##### Whenua (the genealogical or spiritual connection between patient and/or whānau and land)

This relates to the genealogical or spiritual connection between a patient and the land. Many healthy eating programmes identified the connection between whenua and food and the growing and sharing of kai as an important enabler^(34,49,52,53)^. For example, in the OL@-OR@ app, information is provided about historical stories related to food and how to start vegetable gardens^(52)^. Community gardens were also a feature of two intervention programmes^(49,53)^.

##### Whakatere (navigation)

Whakatere or navigation links to best practice in the implementation of interventions^(27)^. Practical support for dietary change was identified as an enabler^(34,41,50)^, and there were several different examples how support was provided. In one study, Kai Manaaki or navigators were specifically employed as support for people with diabetes, both to support them in their interactions with health professionals but also to work to ensure that they had access to all the necessary social welfare support by working with social welfare agencies^(45)^. These enhanced support systems enabled small achievable changes over time^(45)^.

Many studies highlighted that practical support to enable patients to achieve long-term changes included addressing budget and time constraints. Practical strategies included cooking sessions with a focus on healthy, budget-friendly options and meal planning, using traditional foods, gardening workshops, financial advice, budgeting skills, meal planning support, effective nutrition education that included portion sizes and label reading and supermarket tours^(34,35,40,47,49,51,53)^. The practical support did not necessarily need to be provided by dietitians. For example, one intervention worked with practice nurses who were given 6 h training to deliver a support programme for people with prediabetes^(33)^.

##### Personalised clear achievable goals

Mana Motuhake, or the ability to self-determine their own goals, in this case specifically related to healthy food, is essential for Māori from a holistic, well-being perspective. Many studies emphasised the importance of personalised, clear, achievable, stepwise goals^(33,34,45,52)^. For example, in the intervention with practice nurses, individuals with prediabetes worked with nurses to determine three personalised achievable dietary goals, which were recorded in the patient management system for each participant and were then reinforced by general practitioners^(33)^. In the Mana Tū intervention study, goal setting included goals related to a holistic understanding of health, which included social, spiritual and mental well-being^(45)^. Bell *et al.* ^(36)^ also emphasised that goal setting could reconnect people to Indigenous understandings of well-being by facilitating cultural revitalisation through connectedness.

## Discussion

This scoping review examined a range of methodologically diverse literature^(21)^ to identify enablers and barriers to making dietary changes for Māori. Results were summarised and mapped to the Meihana model and illustrated that there is a diverse range of factors influencing dietary change for Māori. The use of the Meihana model as a tool for this analysis is a strength of this review, as it facilitated a culturally appropriate interpretation of the studies, and the model components encapsulated the enablers and barriers for Māori, in a way that is consistent with Māori cultural realities. As the model was designed to support clinical and cultural competence with Māori, not all aspects of the model were necessarily reflected in the research reviewed. Because hauora Māori is viewed holistically, the themes we identified are interconnected and sometimes overlapping.

Many of the barriers identified, such as the cost of food and the difficulty of weight loss, are generic across population groups. However unique to Māori, is the legacy of colonisation on wealth inequity in New Zealand. For example, the median net worth for an NZ European individual was estimated at $151 000 in 2021 compared with $42 000 for Māori^(54)^. In 2017, it was estimated that across an annual year, income inequities result in a total loss to the Māori population of $2·6 billion per year^(55)^. These stark ethnic disparities in wealth and income impact both the financial stress a household faces^(40)^ and the money available for food.

Māori are also more likely to experience racism in the NZ health system^(56)^ and less likely to have a clinician of the same ethnicity; for example, 3·6 % of dietitians are of Māori ethnicity^(57)^. A major inquiry into Māori health noted that the so-called ‘mainstream’ health services often fail to meet Māori health needs and that even when Māori can access mainstream services, often what is being provided simply does not work or is so alienating that people are unable to engage^(5)^.

Māori are a collectivist culture and draw strength within the extended whānau and greater community. Our findings suggest that best practice approaches, grounded in a Māori understanding of well-being, are valued by Māori. This notably included the strength of a collective approach. Many studies included the wider whānau within the intervention, and this contributed to their success^(39,45,53)^. These broader dietary change approaches were often developed using co-design methods and in close conjunction with communities^(39,52,53)^. These processes supported engagement with the interventions and concurrently provided ways to address the impacts of colonisation and racism. Effective co-design requires sustained relationship building and in-depth engagement with the communities affected^(53,58)^.

We also noted that programmes that were informed by a Māori understanding of well-being and worked with participants based on those understandings tended to be more successful than those programmes solely focused on physical goals^(45)^. This reflects the findings by Mack *et al.* ^(18)^, who found in their review on obesity prevention and Māori that key enablers were social connection and a culturally relevant whole-of-life approach informed by Māori models of health^(18)^. Similarly, Korohina *et al.* emphasised and explored the role of matauranga Māori to enable meaningful change for Māori, where weight loss is a positive outcome but not the central goal^(20)^. Goal setting related to physical changes such as weight loss was present in some but not all programmes. This is an important consideration when developing interventions aiming to reduce obesity or related illnesses. Bell *et al.*’s^(36)^ qualitative study of obesity in Indigenous populations found that the sole focus on biomedical markers, caloric restriction, diet and exercise was considered culturally insensitive and was unlikely to support engagement. However, some interventions effectively used goal setting^(28,33,34)^, with emphasis placed on the need for goals to be personally tailored, while taking the wider obesogenic and socio-economic environment into account. More generally, regardless of how interventions were developed, it appears that programmes enabling dietary change involved multidisciplinary input and fostered community involvement and the development of trust. These programmes included multiple intervention components and mechanisms to provide individualised support for change over time. Therefore, a range of programme types, settings and ways of delivering dietary interventions are relevant to Māori, and the assessment of individual preferences is important.

Our findings are relevant to future research and policy. Overall, there was a paucity of research conducted solely within Māori populations, and the relatively low proportion of Māori in several studies may have resulted in Māori views being less apparent. More research with predominantly or solely Māori groups would be helpful. A significant amount of time is needed to establish, refine and embed interventions in real-world settings before the health outcome effects of these interventions can be evaluated. A frustration for health providers involved in programme delivery was short-term funding cycles that prevent programmes from being embedded and achieving change^(48,50)^ or not being able to spend enough time on follow-up with patients^(33)^. This has implications for the funders of programmes and future research, especially where programmes are designed in collaboration with the communities they will serve as long-term funding is required for health benefits to be realised. Research using a range of qualitative and quantitative methodologies needs to be built into the entire duration of such programmes including development (co-design), refinement of programmes and long-term outcome assessment. The significant body of work regarding the Whānau Pakari programme and other work^(48–50,52,53)^ reviewed here provide useful exemplars. Importantly, well-integrated research, which is designed to evaluate what works and why from multiple perspectives, including the programme end users and delivery teams, and is funded within long-term programmes is more likely to result in significant gains in health equity.

These results are relevant for the design of nutrition-related health interventions for Māori. Our findings emphasise the importance of research and programmes that support the enablers of dietary change for Māori, through culturally relevant demedicalised, relational approaches grounded in Māori understandings of health. We note that our findings also correlate with similar research on Indigenous people in other settler colonial states such as Australia^(59)^ and Canada^(60)^. For example, Murdoch-Flowers *et al.*’s^(60)^ work in relation to a Canadian diabetes prevention intervention emphasised the need for culturally based health promotion programmes that collaborate with Indigenous knowledge holders to bring about healthy changes. Similarly, Gwynn *et al.* ^(59)^ found that Indigenous community governance and engagement were a marker of effective nutrition interventions and research with Aboriginal and Torres Strait Islanders in Australia.

Although we aimed to assess the records included in this review for their responsiveness to Māori using the CONSIDER criteria^(26)^, we found much of the data needed for this assessment were missing. As this is a recent framework published later than several of the studies included in this review, this is not surprising. However, future work needs to transparently report how the entire process aligns with best practice for research with Māori or with other Indigenous peoples.

## Conclusion

Using a relevant Indigenous model, this study highlights that multiple and diverse enablers and barriers to dietary change exist for Māori. While some are likely common to all populations, this review highlights the critical importance of developing interventions in close partnership with Indigenous communities, to mitigate the impacts of colonisation and racism and to be grounded in Indigenous understandings of health.

## Supporting information

Mckerchar et al. supplementary material Mckerchar et al. supplementary material

## Figures and Tables

**Table:** 

Māori term^*^	Meaning
Ahua^*^	Personal indicators of Te Ao Māori that are important to the patient/whānau.
Hapū	Subtribe
Hauora	Well-being, health and health practices
Hinengaro^*^	Psychological and emotional well-being
Iwi	Tribe
Kai	Food
Kai manaaki	Community health navigators
Kapa haka	Māori performing arts
Karakia	Incantations
Kaumātua	Māori elder
Kawanatanga	The right of governance
Korikori a Iwi	Study name loosely translated as movement of the people
Mana motuhake	Autonomy, independence, self-determination
Mana tū	Study name meaning to stand with authority
Manaakitanga	The process of showing respect, support and care for others)
Marae	Māori cultural centres
Mātauranga	Traditional knowledge
Mau taiaha	Martial arts
Meihana model	A Māori Indigenous health framework that facilitates fusion of clinical and cultural competencies to better serve Māori
Mokopuna	Grandchild or another related young person
Ngā Hau e Wha^*^	The four winds
Ngā Ratonga Hauora^*^	Health services
Ngā Roma Moana^*^	Ocean currents
Pākehā	Non-Māori, white inhabitants of Aotearoa
Rangatiratanga	Chieftainship, sovereignty, self-determination
Taiao^*^	The physical environment
Tangata whenua	People of the land
Taonga	Treasures
Te Aka Whai Ora	The Māori Health Authority
Te Ao Māori	Māori world view
Te Tiriti o Waitangi	The Treaty of Waitangi
Tikanga	Māori cultural principles
Tinana^*^	Physical health and functioning of the patient
Turangawaewae	Place of belonging
Wairua^*^	Beliefs regarding connectedness and spirituality
Waka ama	Outrigger canoes
Waka hourua^*^	Double-hulled canoe
Whakamā	Shame
Whakamana	Enabling of individuals and families
Whakapapa	Ancestry
Whakatere^*^	Navigation
Whānau ora	Collective family well-being
Whānau Pakari	Study name meaning healthy, self-assured families that are fully active
Whānau^*^	Extended family Within the Meihana model, this term also applies to (a) support networks for the patient, and (b) the relationships, roles and responsibilities of the patient within Te Ao Māori, including whānau, hapu, iwi and other organisations
Whenua^*^	Land. Specific genealogical or spiritual connection between patient and/or whānau and land

* This term is used in the Meihana model, and definitions align with the way they are used in this model.

## Data Availability

All data used in this review are available in previously published papers.

## Supplementary material

For supplementary material accompanying this paper, visit https://doi.org/10.1017/S136898002400212X.
