# Supplementary material for: Enablers and barriers to dietary change for Māori with nutrition-related conditions in Aotearoa New Zealand: a scoping review
Source: Public Health Nutr. 2024 Oct 22;27(1):e245. doi: 10.1017/S136898002400212X (PMC11705027; doi:10.1017/S136898002400212X)
Supplement: Mckerchar et al. supplementary material [file S136898002400212Xsup001.docx]

**Enablers and barriers to dietary change for Māori with nutrition-related conditions in Aotearoa, New Zealand: a scoping review**

## Supplementary files

### Supplementary Table 1: Preferred Reporting Items for Systematic reviews and Meta-Analyses extension for Scoping Reviews (PRISMA-ScR) Checklist

| SECTION | ITEM | PRISMA-ScR CHECKLIST ITEM | REPORTED ON PAGE # |
| --- | --- | --- | --- |
| TITLE | | | |
| Title | 1 | Identify the report as a scoping review. | Page 1 |
| ABSTRACT | | | |
| Structured summary | 2 | Provide a structured summary that includes (as applicable): background, objectives, eligibility criteria, sources of evidence, charting methods, results, and conclusions that relate to the review questions and objectives. | Page 1-2 |
| INTRODUCTION | | | |
| Rationale | 3 | Describe the rationale for the review in the context of what is already known. Explain why the review questions/objectives lend themselves to a scoping review approach. | Page 3-4 |
| Objectives | 4 | Provide an explicit statement of the questions and objectives being addressed with reference to their key elements (e.g., population or participants, concepts, and context) or other relevant key elements used to conceptualise the review questions and/or objectives. | Page 4 & Figure 1 |
| METHODS | | | |
| Protocol and registration | 5 | Indicate whether a review protocol exists; state if and where it can be accessed (e.g., a Web address); and if available, provide registration information, including the registration number. | Page 4, line 94 |
| Eligibility criteria | 6 | Specify characteristics of the sources of evidence used as eligibility criteria (e.g., years considered, language, and publication status), and provide a rationale. | Figure 1 |
| Information sources* | 7 | Describe all information sources in the search (e.g., databases with dates of coverage and contact with authors to identify additional sources), as well as the date the most recent search was executed. | Page 1, Table 1 |
| Search | 8 | Present the full electronic search strategy for at least 1 database, including any limits used, such that it could be repeated. | Supplementary Table 2 |
| Selection of sources of evidence† | 9 | State the process for selecting sources of evidence (i.e., screening and eligibility) included in the scoping review. | Table 1, Figure 1 |
| Data charting process‡ | 10 | Describe the methods of charting data from the included sources of evidence (e.g., calibrated forms or forms that have been tested by the team before their use, and whether data charting was done independently or in duplicate) and any processes for obtaining and confirming data from investigators. | Table 1 |
| Data items | 11 | List and define all variables for which data were sought and any assumptions and simplifications made. | Tables 1 and 3 |
| Critical appraisal of individual sources of evidence§ | 12 | If done, provide a rationale for conducting a critical appraisal of included sources of evidence; describe the methods used and how this information was used in any data synthesis (if appropriate). | Page 4, Table 1 |
| Synthesis of results | 13 | Describe the methods of handling and summarising the data that were charted. | Table 1, 3 |
| RESULTS | | | |
| Selection of sources of evidence | 14 | Give numbers of sources of evidence screened, assessed for eligibility, and included in the review, with reasons for exclusions at each stage, ideally using a flow diagram. | Figure 3 |
| Characteristics of sources of evidence | 15 | For each source of evidence, present characteristics for which data were charted and provide the citations. | Table 3 |
| Critical appraisal within sources of evidence | 16 | If done, present data on critical appraisal of included sources of evidence (see item 12). | Table 3 |
| Results of individual sources of evidence | 17 | For each included source of evidence, present the relevant data that were charted that relate to the review questions and objectives. | Pages 6-13, Table 3 |
| Synthesis of results | 18 | Summarise and/or present the charting results as they relate to the review questions and objectives. | Pages 6-13 |
| DISCUSSION | | | |
| Summary of evidence | 19 | Summarise the main results (including an overview of concepts, themes, and types of evidence available), link to the review questions and objectives, and consider the relevance to key groups. | Page 13-15 |
| Limitations | 20 | Discuss the limitations of the scoping review process. | Page 15 |
| Conclusions | 21 | Provide a general interpretation of the results with respect to the review questions and objectives, as well as potential implications and/or next steps. | Page 15 |
| FUNDING | | | |
| Funding | 22 | Describe sources of funding for the included sources of evidence, as well as sources of funding for the scoping review. Describe the role of the funders of the scoping review. | Title page |

JBI = Joanna Briggs Institute; PRISMA-ScR = Preferred Reporting Items for Systematic reviews and Meta-Analyses extension for Scoping Reviews.

* Where *sources of evidence* (see second footnote) are compiled from, such as bibliographic databases, social media platforms, and Web sites.

† A more inclusive/heterogeneous term used to account for the different types of evidence or data sources (e.g., quantitative and/or qualitative research, expert opinion, and policy documents) that may be eligible in a scoping review as opposed to only studies. This is not to be confused with *information sources* (see first footnote).

‡ The frameworks by Arksey and O’Malley (6) and Levac and colleagues (7) and the JBI guidance (4, 5) refer to the process of data extraction in a scoping review as data charting*.*

§ The process of systematically examining research evidence to assess its validity, results, and relevance before using it to inform a decision. This term is used for items 12 and 19 instead of "risk of bias" (which is more applicable to systematic reviews of interventions) to include and acknowledge the various sources of evidence that may be used in a scoping review (e.g., quantitative and/or qualitative research, expert opinion, and policy document).

*From:* Tricco AC, Lillie E, Zarin W, O'Brien KK, Colquhoun H, Levac D, et al. PRISMA Extension for Scoping Reviews (PRISMAScR): Checklist and Explanation. Ann Intern Med. 2018;169:467–473. [doi: 10.7326/M18-0850](http://annals.org/aim/fullarticle/2700389/prisma-extension-scoping-reviews-prisma-scr-checklist-explanation).

### Supplementary Table 2: Search Strategies

Search Strategy Scoping Review – all searches undertaken 27 07 2021

| **Data base** | **Search strategy** |
| --- | --- |
| Embase | 1. pacific islands/ or exp melanesia/ or exp micronesia/ or exp polynesia/ 2. (melanesia* or micronesia* or polynesia*).mp. 3. (aotearoa* or maori* or maaori* or samoa* or fiji* or tahiti* or "the marianas" or (new adj caledoni*) or rarotonga* or marquesas or tuamotu* or kiriba* or nauru* or niue* or palau* or papua* or (new adj guinea*) or tuvalu* or tonga* or tokelau* or vanuatu* or chuuk* or kosrae* or pohnpei* or yap* or guam or pitcairn* or futun* or marianna* or hawaii* or wallisian or chamorro* or guamanian or gilbertese or belau or ((caroline or cook or windward or leeward or austral or society or gambier or gilbert or ellice or marshall or easter or solomon or soloman or wallis or mariana or pacific) adj island*) or (kingdom adj uvea)).ti,ab,kw. 4. oceanic ancestry group/ 5. ((pacific adj3 (island* or people*)) or "new zealand born pacific*").ti,ab,kw. 6. (pacifica* or pasifika* or pasefik*).ti,ab,kw. 7. 1 or 2 or 3 or 4 or 5 or 6 8. exp diet/ 9. (food* or diet* or nutri* or eat* or meal*).ab. /freq=2 10. nutrition policy/ 11. nutrition therapy.mp. or exp diet therapy/ 12. eating/ 13. nutrition/ or food/ 14. 8 or 9 or 10 or 11 or 12 or 13 15. feeding behavior/ or food preference/ 16. exp attitude to health/ or habits/ or harm reduction/ or health behavior/ or personal satisfaction/ or social behavior/ or ceremonial behavior/ or empowerment/ or mass behavior/ or self-control/ or social conformity/ or social facilitation/ or exp motivation/ or exp personality/ 17. (attitude* or accept* or barrier* or facilitat* or challeng* or preference* or motivat* or satisf* or knowledge* or knowing or behavi* or literac* or understand* or habit or habits or uptake or adopt* or comply* or compliance or complied or influen* or cultur* or sociocultur* or inform* or Self-efficac* or partnership or Availibil* or responsibil*).ti,ab,kw. 18. 15 or 16 or 17 19. (intervention* or program* or therap* or initiative* or plan* or treatment* or schedul* or management or manage or managing or prevention or preventive or "primary prevention" or "secondary prevention").mp. 20. 7 and 14 and 18 21. 19 and 20 22. limit 21 to english language 23. limit 22 to human |
| Medline | 1. pacific islands/ or exp melanesia/ or exp micronesia/ or exp polynesia/ or new zealand/ 2. (melanesia* or micronesia* or polynesia* or "new zealand*").mp. 3. (aotearoa* or maori* or maaori* or samoa* or fiji* or tahiti* or "the marianas" or (new adj caledoni*) or rarotonga* or marquesas or tuamotu* or kiriba* or nauru* or niue* or palau* or papua* or (new adj guinea*) or tuvalu* or tonga* or tokelau* or vanuatu* or chuuk* or kosrae* or pohnpei* or yap* or guam or pitcairn* or futun* or marianna* or hawaii* or wallisian or chamorro* or guamanian or gilbertese or belau or ((caroline or cook or windward or leeward or austral or society or gambier or gilbert or ellice or marshall or easter or solomon or soloman or wallis or mariana or pacific) adj island*) or (kingdom adj uvea)).tw,kw,kf. 4. Oceanic Ancestry Group/ 5. ((pacific adj3 (island* or people*)) or "new zealand born pacific*").tw,kw,kf. 6. (pacifica* or pasifika* or pasefik*).tw,kw,kf. 7. 1 or 2 or 3 or 4 or 5 or 6 8. exp Diet/ 9. (food* or diet* or nutri* or eat* or meal*).ti,kw,kf. or (food* or diet* or nutri* or eat* or meal*).ab. /freq=2 10. Nutrition Policy/ 11. nutrition therapy/ or exp diet therapy/ 12. Eating/ 13. exp *"diet, food, and nutrition"/ 14. 8 or 9 or 10 or 11 or 12 or 13 15. feeding behavior/ or food preferences/ 16. exp attitude to health/ or habits/ or harm reduction/ or health behavior/ or personal satisfaction/ or social behavior/ or ceremonial behavior/ or empowerment/ or mass behavior/ or self-control/ or social conformity/ or social facilitation/ or exp motivation/ or exp personality/ 17. (attitude* or accept* or barrier* or facilitat* or challeng* or preference* or motivat* or satisf* or knowledge* or knowing or behavi* or literac* or understand* or habit or habits or uptake or adopt* or comply* or compliance or complied or influen* or cultur* or sociocultur* or inform* or Self-efficac* or partnership or Availibil* or responsibil*).ti,kw,kf. 18. 15 or 16 or 17 19. (intervention* or program* or therap* or initiative* or plan* or treatment* or schedul* or management or manage or managing or prevention or preventive).mp. 20. 7 and 14 and 18 21. 19 and 20 22. limit 21 to english language 23. limit 22 to "humans only (removes records about animals)" |
| Scopus | 1. "pacific island*" OR "melanesia*" OR "micronesia*" OR polynesia* OR "aotearoa" OR "maori" OR "maaori" OR "samoa* "OR "fiji*" OR "tahiti*" OR "the marianas" OR "new caledoni*" OR "rarotonga*" OR "marquesas" OR "tuamotu*" OR "kiriba*" OR "nauru*" OR "niue*" OR "palau*" OR "tuvalu*" OR "tonga*" OR "tokelau*" OR "vanuatu*" OR "chuuk*" OR "kosrae*" OR "pohnpei*" OR "yap*" OR "pitcairn*" OR "futun*" OR "marianna*" OR "wallisian" OR "gilbertese" OR "belau" OR (island W/3 (caroline OR cook OR windward OR leeward OR austral OR society OR gambier OR gilbert OR ellice OR marshall OR easter OR solomon OR soloman OR wallis OR mariana OR "kingdom uvea")) OR "oceanic ancestry group" (pacific W/3 (island* OR people*)) OR "new zealand born pacific*" OR "pacifica*" OR "pasifika*" OR "pasefik*" 2. diet OR food* OR nutri* OR eat* OR meal* OR "nutrition policy" OR "nutrition therapy" OR "diet therapy" OR eating OR "diet, food, and nutrition" 3. “feeding behav*” OR “food preference” OR “attitude to health” OR “habits” OR “harm reduction” OR “health behav*” OR “personal satisfaction” OR “social behav*” OR “ceremonial behav*” OR “empowerment” OR “mass behav*” OR “self-control” OR “social conformity” OR “social facilitation” OR “motivation” OR “personality” OR “attitud*” OR “accept*” OR “barrier*” OR “facilitat*” OR “challeng*” OR “preference*” OR “motivat*” OR “satisf*” OR “knowledge*” OR “knowing” OR “behavi*” OR “literac*” OR “understand*” OR “habit” OR “habits” OR “uptake” OR “adopt*” OR “comply*” OR “compliance” OR “complied” OR “influenc*” OR “cultur*” OR “sociocultur*” OR “inform*” OR “self-effic*” OR “partnership” OR “availibil*” OR “responsibil*” |
| NZ Research | 1. (diet* OR food* OR nutri* OR eat* OR meal*) AND (pacific* OR samoa* OR fiji* OR maori* OR tonga* OR maaori* OR polynesia* OR aotearoa OR tahiti* OR "cook island*" OR rarotonga* OR kiriba* OR niue* OR tuvalu* OR tokelau* OR pacifica* OR pasifika* OR pasefik* OR melanesia* OR micronesia* OR "new zealand born pacific*") AND (“feeding behav*” OR “food preference” OR “attitude to health” OR “habits” OR “harm reduction” OR “health behav*” OR “personal satisfaction” OR “social behav*” OR “ceremonial behav*” OR “empowerment” OR “mass behav*” OR “self-control” OR “social conformity” OR “social facilitation” OR “motivation” OR “personality” OR “attitud*” OR “accept*” OR “barrier*” OR “facilitat*” OR “challeng*” OR “preference*” OR “motivat*” OR “satisf*” OR “knowledge*” OR “knowing” OR “behavi*” OR “literac*” OR “understand*” OR “habit” OR “habits” OR “uptake” OR “adopt*” OR “comply*” OR “compliance” OR “complied” OR “influenc*” OR “cultur*” OR “sociocultur*” OR “inform*” OR “self-effic*” OR “partnership” OR “availibil*” OR “responsibil*”) |
| Informit | 1. diet* OR food* OR nutri* OR eat* OR meal* 2. (pacific* OR samoa* OR fiji* OR maori* OR tonga* OR maaori* OR polynesia* OR aotearoa OR tahiti* OR "cook island*" OR rarotonga* OR kiriba* OR niue* OR tuvalu* OR tokelau* OR pacifica* OR pasifika* OR pasefik* OR melanesia* OR micronesia* OR "new zealand born pacific*") |
